# Preparation of Low Molecular Weight Chondroitin Sulfates, Screening of a High Anti-Complement Capacity of Low Molecular Weight Chondroitin Sulfate and Its Biological Activity Studies in Attenuating Osteoarthritis

**DOI:** 10.3390/ijms17101685

**Published:** 2016-10-11

**Authors:** Lian Li, Yan Li, Danyang Feng, Linghua Xu, Fengxin Yin, Hengchang Zang, Chunhui Liu, Fengshan Wang

**Affiliations:** 1 Key Laboratory of Chemical Biology of Natural Products (Ministry of Education), Institute of Biochemical and Biotechnological Drug, School of Pharmaceutical Sciences, Shandong University, No. 44 Wenhuaxi Road, Jinan 250012, China; doublel3314@126.com (L.L.); liyan0922@hotmail.com (Y.L.); angelic1990@163.com (D.F.); xulinghua09199@163.com (L.X.); xin63518@163.com (F.Y.); zanghcw@126.com (H.Z.); liuchunhui@sdu.edu.cn (C.L.); 2 National Glycoengineering Research Center, Shandong University, Jinan 250012, China

**Keywords:** chondroitin sulfate, anti-complement, osteoarthritis, depolymerization

## Abstract

Chondroitin sulfate (CS) plays important roles in the complement system. However, the CS structure is complicated due to different sources and the number and positions of sulfate groups. The objective of this study was to prepare different low molecular weight chondroitin sulfates (LMWCSs) and to investigate the biological activity in anti-complement capacity. A series of LMWCSs was prepared from different sources and characterized by ultraviolet-visible (UV) spectroscopy, high-performance liquid chromatography (HPLC), size exclusion chromatography-multiangle laser light scattering (SEC-MALLS) and nuclear magnetic resonance (NMR) spectroscopy. Hemolytic, anti-complement 3 deposition capacity and cell viability assays were carried out to investigate the biological activities in vitro. The results showed that LMWCS prepared from shark cartilage with the oxidative degradation method (LMWCS-S-O) had the best anti-complement capacity. LMWCS-S-O could inhibit the alternative pathway of the complement system and protect chondrocytes from cell death. The attenuating effect of LMWCS-S-O on Osteoarthritis (OA) was investigated by destabilization of the medial meniscus (DMM) model in vivo. Functional wind-up, histological and C5b-9 analyses were used to evaluate the treatment effect on the OA model. In vivo results showed that LMWCS-S-O could attenuate OA. LMWCS-S-O with a high content of ΔDi-2,6diS and ΔDi-6S could be used for attenuating OA through regulating the complement system.

## 1. Introduction

Osteoarthritis (OA) is a disease caused by both mechanical and biological degradation of cartilage, especially in old people [1]. Although the exact mechanisms are not well understood, many data showed that the pathogenesis of OA is attributed to the immune system [2], among which the complement system plays key roles [3]. The complement system is composed of three activation pathways, the classical pathway, alternative pathway and lectin pathway [4]. Additionally, the alternative pathway is the main cause for OA [5]. Therefore, complement regulators in the alternative pathway may have great potential in the prevention and treatment of OA.

Glycosaminoglycans (GAGs) play important roles in the complement system [6]. Heparin was first found to have good anti-complement activity in 1929 [7]. Heparan sulfate (HS) could inhibit the complement alternative pathway, which indicates that HS plays a key role in the transition between normal aging and age-related macular degeneration [8]. CS is also reported to have biological activities in the complement system [9]. However, due to the difference in structure, sources and molecular weight, CS has complicated biological activities in the complement system [10]. CS is composed alternatively of the disaccharide unit d-glucuronic acid (GlcA) and *N*-acetyl-d-galactosamine (GalNAc) residues. [11,12]. Based on the number and positions of sulfate groups, there are about 25 CS disaccharides [12]. CSs are commonly sulfated at the C-4 and/or C-6 of GalNAc in mammals, which are chondroitin-4-sulfate and chondroitin-6-sulfate. The CS structure varies with different sources, even different tissues and ages in the same source [13]. CS on the market is usually from shark, porcine and bovine cartilage [14]. A previous study in our laboratory indicated that the disulfated disaccharide D (GlcA(2S)-GalNAc(6S)) and E (GlcA-GalNAc(4S,6S)) units are the characteristic components in shark cartilage, and the main composition in shark cartilage is chondroitin-6-sulfate, while chondroitin-4-sulfate is the main composition of CS from porcine and bovine cartilage [11]. The result was in accordance with the data published by Volpi [15]. The molecular weight of CS also affects its bioactivity. It is difficult for intact CS to pass through the gastric and intestinal mucosa [16]. Low molecular weight chondroitin sulfate (LMWCS) could be absorbed through the gastrointestinal tract [17,18].

In the present study, LMWCSs were prepared by the degradation of shark, porcine and bovine cartilage CSs using four different methods. The resulting LMWCSs were characterized using ultraviolet-visible (UV) spectroscopy, high performance liquid chromatography (HPLC), size exclusion chromatography-multiangle laser light scattering (SEC-MALLS) and nuclear magnetic resonance (NMR) spectroscopy. The hemolytic assay was employed to evaluate their anti-complement activities in vitro. Then, the anti-complement 3 deposition capacity and cell viability assays were used to further evaluate the anti-complement capacity. The destabilization of the medial meniscus (DMM) OA animal model was established to evaluate the prevention and treatment effect of LMWCS on OA as a complement activity inhibitor in vivo. This study indicated that LMWCS had OA attenuating potential via inhibiting the activity of complements.

## 2. Results

### 2.1. Preparation and Characterization of Low Molecular Weight Chondroitin Sulfate (LMWCSs)

**Figure 1:**
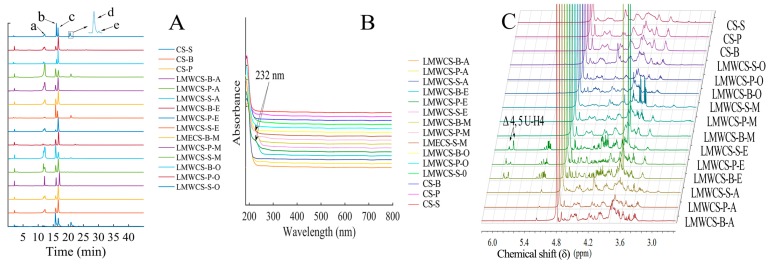
Characterization of low molecular weight chondroitin sulfate (LMWCS) samples. (**A**) Disaccharide compositions of all CS samples from three sources depolymerized by different methods. B, P and S stand for bovine, porcine and shark cartilage, respectively. A stands for the HCl method; E stands for enzymatic method; M stands for microwave-assisted alkaline method; and O stands for oxidative method. a, ΔDi-0S; b, ΔDi-6S; c, ΔDi-4S; d, ΔDi-2,6diS; e, ΔDi-4,6diS; (**B**) UV spectra of all CS samples; (**C**) Proton nuclear magnetic resonance (^1^H-NMR) spectra of chondroitin sulfates (CSs) from three sources and low molecular weight chondroitin sulfates (LMWCSs) depolymerized by different methods.

In order to get different LMWCSs, four different methods were used to prepare LMWCSs from shark, porcine and bovine cartilage. LMWCSs were characterized by UV, HPLC, NMR (Figure 1) and SEC-MALLS. Figure 1A shows the CS chromatograms. All samples were composed mainly of chondroitin-6-sulfate and chondroitin-4-sulfate. CS and its depolymerized products from shark cartilage have chondroitin-2,6-sulfate and chondroitin-4,6-sulfate, while the other two sources did not. The disaccharide compositions of all CS samples are shown in Table 1. The results in Table 1 show that different degradation methods led to diverse sulfate contents. UV spectroscopy was employed to investigate the effects caused by different methods. Figure 1B showed that LMWCSs from enzymatic methods had significant absorption at 232 nm, which indicated the formation of Δ4,5-unsaturated disaccharides, while other samples did not show significant absorption. The ${\bar{M}}_{W}$ is shown in Table 1. The ${\bar{M}}_{W}$ of LMWCSs ranges from 1217 to 8085 Da. NMR was also employed to investigate the structure of LMWCSs. LMWCSs from enzymatic methods showed additional chemical shifts at approximately 5.18, 3.78, 4.10 and 5.88 ppm for the H-1, H-2, H-3 and H-4 protons, respectively, indicating a non-reducing terminal Δ4,5-unsaturated uronic acid (ΔUA) residue, and the results were in accordance with references [19,20]. The major chemical shifts of LMWCSs from the remaining prepared methods were consistent with those of intact CS, which indicated that the non-reducing and reducing end were the same as intact CS. The results were the same as Cho and coworkers’ results [18].

### 2.2. In Vitro Effect of LMWCSs on Complement Activities

#### 2.2.1. Hemolytic Analysis

The anti-complement activities of CS and all 12 LMWCSs were evaluated in vitro by the hemolytic micro assay. The results are shown in Table 2. LMWCS prepared from shark cartilage with the oxidative degradation method (LMWCS-S-O) had the highest anti-complement capacity, while CS from porcine cartilage (CS-P) had the lowest activity. Therefore, LMWCS-S-O and CS-P were used to investigate the anti-complement activity further.

#### 2.2.2. Anti-C3 Deposition Capacity

**Figure 2:**
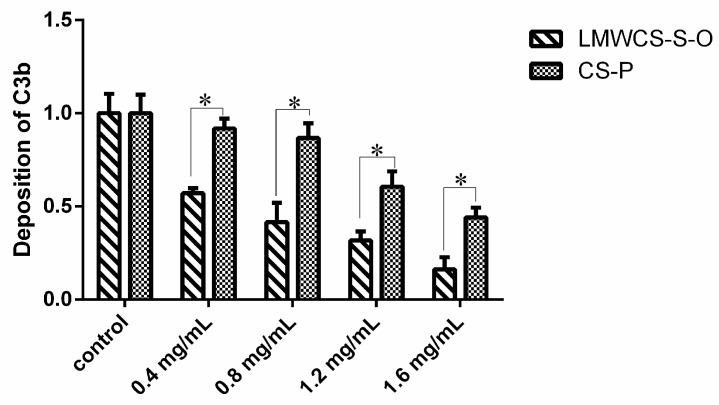
Effect of LMWCS-S-O and CS-P on the alternative pathway of the complement system. All data were normalized by setting the absorbance of the control group to one. (* *p* < 0.05 vs. CS-P groups).

The inhibitory effect of LMWCS-S-O and CS-P on C3 deposition in the alternative pathway was investigated using the ELISA method. The addition of different concentrations of LMWCS-S-O and CS-P resulted in the decreased amounts of C3b in a dose-dependent manner (Figure 2). All LMWCS-S-O groups showed a significant decrease compared to the control group, while only CS-P groups at 1.2 mg/mL and 1.6 mg/mL showed a significant decrease compared to the control group. All LMWCS-S-O groups showed a significantly higher inhibitory effect compared to CS-P groups in the same concentrations. The results indicated that LMWCS-S-O had better anti-complement activity in the alternative pathway.

#### 2.2.3. Cell Viability Analysis

**Figure 3:**
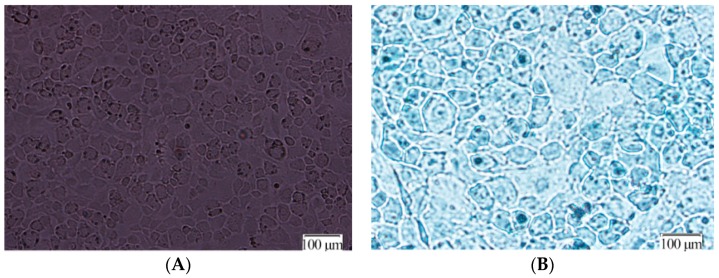
Phenotyping of articular chondrocytes. (**A**) The chondrocyte morphology with a rounded or polygonal shape; (**B**) the presence of sulfated proteoglycans.

Articular chondrocytes were isolated and the morphological aspects were investigated in order to identify chondrocytes. The results are shown in Figure 3. No dead cells were observed with a light-contrast microscope using the trypan blue method. Articular chondrocytes exhibited a rounded and polygonal typical shape (Figure 3A), and sulfated proteoglycans were stained by alcian blue (Figure 3B).

**Figure 4:**
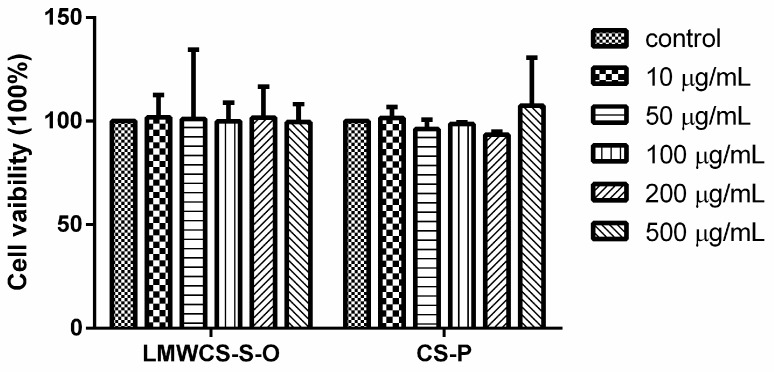
Cytotoxic effect of LMWCS-S-O and CS-P on articular chondrocytes after the treatment with LMWCS-S-O and CS-P for 24 h.

The cytotoxic effect of LMWCS-S-O and CS-P on the articular chondrocytes was determined using the MTT assay. The results in Figure 4 showed that both LMWCS-S-O and CS-P at 10, 50, 100, 200 and 500 μg/mL did not show a cytotoxic effect on chondrocytes after 24 h on articular chondrocytes. Hence, the effect of LMWCS-S-O and CS-P on the cells in the following study could be omitted.

**Figure 5:**
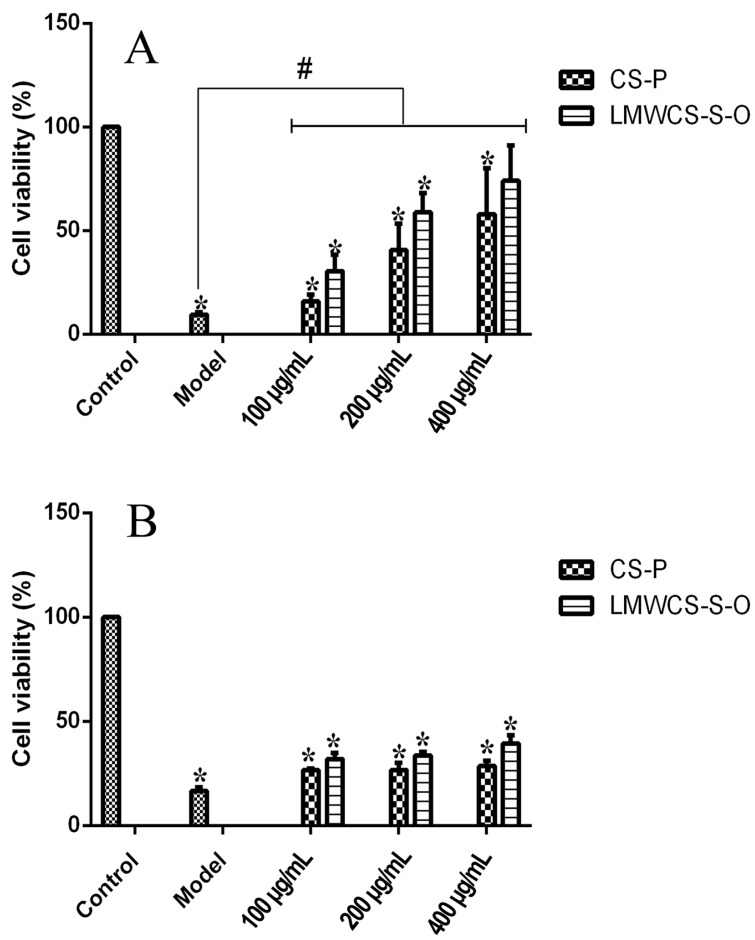
Articular chondrocytes cell death mediated by normal human serum (NHS) at 12 h (**A**); and 24 h (**B**), respectively. The LMWCS-S-O group at 400 μg/mL did not show a significant difference from the control group at 12 h. The cell viabilities of all the other groups were significantly lower compared to the control group. All groups showed a protective effect compared to the model group (* *p* < 0.05 vs. the control group, ^#^ *p* < 0.05 vs. the model group).

In order to investigate the protective ability of LMWCS-S-O and CS-P to the chondrocytes in the complement system, various concentrations of LMWCS-S-O and CS-P mixed with 20% normal human serum (NHS) were added into the wells of 96-well plate and incubated for 12 and 24 h, respectively. The results are shown in Figure 5. Different degrees of cell death were obtained with 20% NHS compared to the control group at 12 h and 24 h, respectively. One hundred, 200 and 400 μg/mL of LMWCS-S-O and CS-P samples were added into the NHS to interact with the complements. The LMWCS-S-O group at 400 μg/mL did not show significant difference from the control group at 12 h. The cell viabilities of all of the other dose groups were significantly lower compared to the control group at 12 h. The results indicated that LMWCS-S-O at 400 μg/mL could protect chondrocytes from lysis by NHS. However, the cell viabilities of all LMWCS-S-O and CS-P groups were significantly higher compared to the model group at 12 h. The results in 24 h indicated that both samples had slightly protective effects on the chondrocytes compared to the model group. The LMWCS-S-O group had a better protection effect than the CS-P group at 24 h. All results demonstrated that LMWCS-S-O protects the chondrocytes from cell death caused by the complements.

### 2.3. In Vivo Effect of LMWCSs on the OA Model

#### 2.3.1. Functional Wind-up Determinations

**Figure 6:**
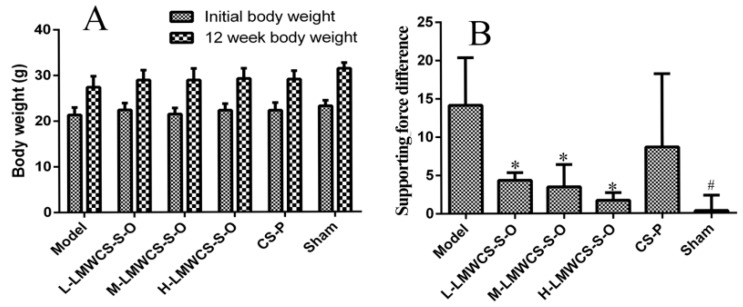
Physical characteristic results of the experiment animals. (**A**) The body weights of all six groups initially and at 12 weeks; (**B**) functional wind-up analysis of all mice administered with LMWCS-S-O or CS-P, where the L-LMWCS-S-O, M-LMWCS-S-O and H-LMWCS-S-O groups were administered with 50, 150 and 450 mg/kg of LMWCS-S-O, respectively. The CS-P group was administered with 150 mg/kg of CS-P. The sham and model groups were administered with saline once a day. * *p* < 0.05 vs. the model, ^#^ *p* < 0.05 vs. the model.

Both bodyweights and functional wind-up (FWU) results are shown in Figure 6. The body weights increased for all groups after 12 weeks, and the weights in the model group were a little lower than the other groups after 12 weeks. FWU was carried out to investigate the physical characteristic of OA. Compared to the model group, the supporting force difference between the left and right hind legs of the low dose group (L-LMWCS-S-O), middle dose group (M-LMWCS-S-O) and high dose group (H-LMWCS-S-O) was significantly lower, which meant that the mice administered with LMWCS-S-O orally had a curing effect on OA. While the supporting force difference of the CS-P group did not show a significant difference from the model group, the data of the CS-P group looked lower than that of the model group.

#### 2.3.2. Histological Analysis

**Figure 7:**
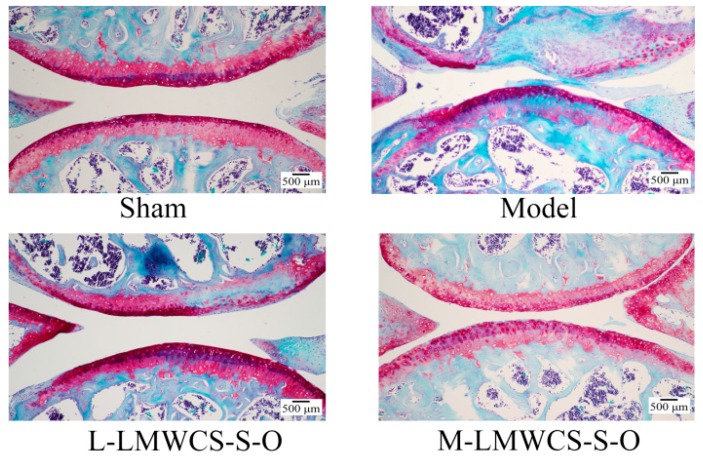

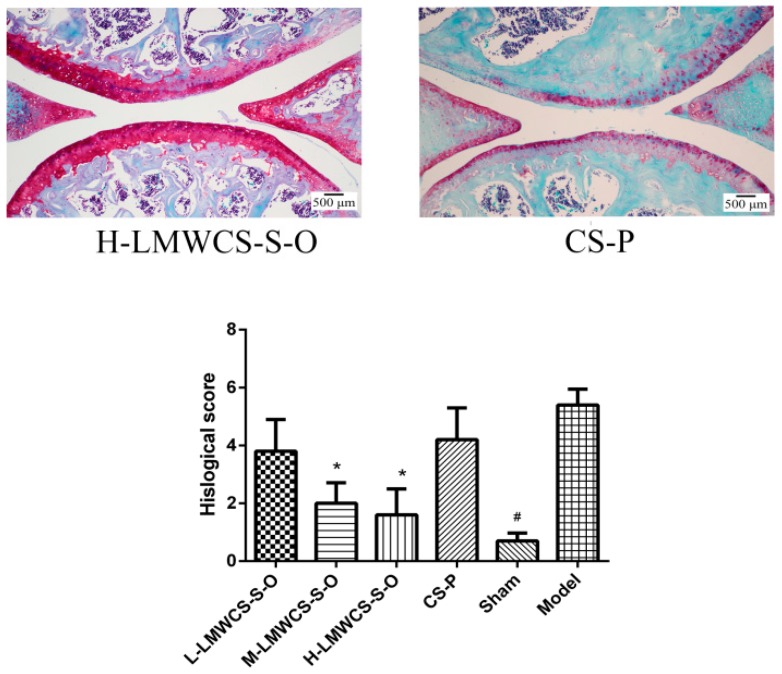
Histological analysis of the joint cartilage and semi-quantitative OA grade analysis. Semi-quantitative OA grade analysis presented in bar graphs (*n* ≥ 5). * *p* < 0.05 vs. the model; ^#^ *p* < 0.05 vs. the model. The L-LMWCS-S-O, M-LMWCS-S-O and H-LMWCS-S-O groups were administered with 50, 150 and 450 mg/kg of LMWCS-S-O, respectively. The CS-P group was administered with 150 mg/kg of CS-P.

At 12 weeks post-operative, all mice were sacrificed for histological analysis, and the results are shown in Figure 7. Safranin-O-stained cartilage showed that fibrillation and erosions could be found in the DMM model group, and about 50% Safranin-O was lost in the model group. In contrast, the sham group showed no structural lesions and little Safranin-O loss. Compared with the model group, the low dose group (L-LMWCS-S-O) showed no structural lesions, but about 10% Safranin-O loss. The L-LMWCS-S-O group did not show a significant difference from the model group. The middle dose LMWCS-S-O (M-LMWCS-S-O) and high dose LMWCS-S-O (H-LMWCS-S-O) groups significantly attenuated articular cartilage erosion compared to the model group. The cartilage structure was intact, and little Safranin-O loss was found. However, the CS-P group did not show a significance difference from the model group, though it showed some improvement compared to the model group. The in vivo results demonstrated that LMWCS-S-O had a higher protective effect compared to CS-P.

#### 2.3.3. Levels of C5b-9 in Serum

**Figure 8:**
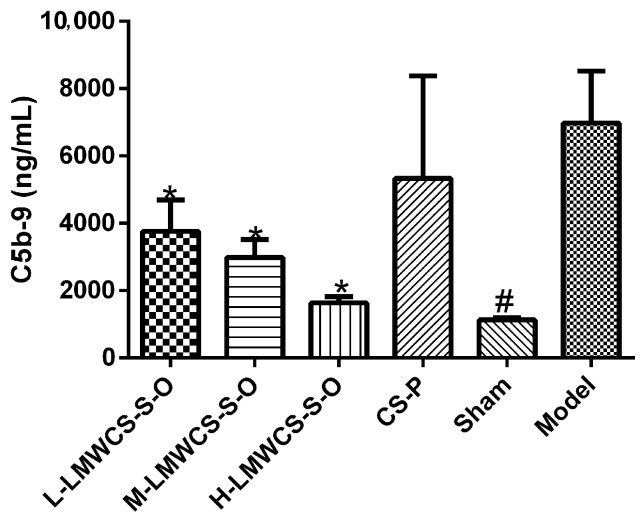
C5b-9 levels of all mice administered with LMWCS-S-O and CS-P. The L-LMWCS-S-O, M-LMWCS-S-O and H-LMWCS-S-O groups were administered with 50, 150 and 450 mg/kg of LMWCS-S-O, respectively. The CS-P group was administered with 150 mg/kg of CS-P. The sham and model groups were administered with saline. Bar graphs present *n* ≥ 5. * *p* < 0.05 vs. the model; ^#^ *p* < 0.05 vs. the model.

C5b-9 levels were determined using the ELISA method, and the result is shown in Figure 8. Compared with the model group, the C5b-9 levels in all LMWCS-S-O groups were significantly lower than in the model group. The C5b-9 level in the CS-P group did not show a significant difference from the model group, though the data of the CS-P group looked lower than that of the model group.

## 3. Discussion

The physical and chemical properties of LMWCSs could be affected by different sources and degradation methods. HPLC was used to investigate the disaccharides of LMWCSs, which indicated that LMWCS from shark cartilage had chondroitin-2,6-sulfate and chondroitin-4,6-sulfate, which were the characteristic components in shark cartilage. HCl and the microwave-assisted alkaline method caused significant loss of the sulfate contents. The oxidative degradation method had a less significant effect on sulfate content and disaccharide composition with respect to acid and the microwave-assisted alkaline method. The NMR results showed that the non-reducing and reducing ends of LMWCSs from oxidative, HCl and microwave-assisted alkaline methods were the same as intact CSs, which were in accordance with [18]. The enzymatic method was gentle, but it caused the formation of ΔUA residue at the non-reducing end, which was shown in Figure 1C. The anti-complement activity had a close relationship with the CS compositions and structure. Therefore, the anti-complement activity of all CS and LMWCS samples was investigated by hemolytic analysis. Generally, compared with different sources, the LMWCSs from shark cartilage had better activity than those from porcine and bovine cartilage. This may be caused by the sulfation positions. Chondroitin-2,6-sulfate existed in shark cartilage CS, but not in the other two sources, and the content of chondroitin-6-sulfate was higher in shark cartilage CS than that in the other two sources. The results indicated that disulfation CS and chondroitin-6-sulfate might play important roles in inhibiting complement activity, and the result was similar to Skliris’ results, which indicated that chondroitin-6-sulfate and chondroitin-2,6-sulfate had higher anti-complement activity than chondroitin-4-sulfate [10]. The anti-complement activity of LMWCS-S-E was not as good as LMWCS-S-O; this may be caused by the formation of the Δ4,5-unsaturated uronic acid and the different complement at which we are looking. The biological activities among different preparation methods were compared. LMWCSs prepared by the oxidative degradation method had better activity than that by other three degradation methods. HCl and microwave-assisted degradation led to the loss of sulfate contents, which was important for the anti-complement activity of CS. In the enzymatic method, Δ4,5-unsaturated disaccharides were formed (Figure 1B), and the NMR results also indicated that the ΔUA was the non-reducing end for LMWCSs from enzymatic methods, which may decrease the anti-complement activity. The anti-complement activity of LMWCSs was better than that of non-degraded CS generally. Hence, LMWCS-S-O with a high content of chondroitin 6-sulfate and chondroitin-2,6-sulfate had higher anti-complement activity. Therefore, LMWCS-S-O with higher anti-complement activity and CS-P with lower activity were used for the following in vitro and in vivo studies.

The alternative pathway can be activated by danger-associated molecular patterns (DAMPs), including cartilage matrix constituents, apoptotic cells or debris of dead cells [21]. Then, C3 is hydrolyzed into C3b, which causes the generation of C5a and the membrane attack complex (MAC, also named C5b-9) [22]. C5b-9 could cause the destruction of the cartilage matrix and chondrocytes lysis, which might cause OA finally. Therefore, the anti-C3 deposition capacity in the alternative pathway was studied. LMWCS-S-O had better bioactivity; this may be due to the high content of ΔDi-6S, ΔDi-2,6diS and low ${\bar{M}}_{W}$. Skliris et al. demonstrated that the anti-complement activity of chondroitin-2,6-sulfate and chondroitin-6-sulfate was better than that of chondroitin-4-sulfate [10,21].

The protective ability of LMWCS-S-O and CS-P for the chondrocytes in the complement system was investigated. Primary articular chondrocytes were used in this study, because articular chondrocytes would lose their differentiated phenotype upon repeated passages and the response to IL-1β would decrease [23]. MTT results showed that LMWCS-S-O had no cytotoxic effect, which is in accordance with the results of Xiao et al. [17]. Cell death mediated by NHS could be used to evaluate the protective ability to chondrocytes [24]. Hence, in this study, the protective ability of LMWCS-S-O and CS-P for the chondrocytes was investigated by this method. The results showed that LMWCS-S-O could interact with complement proteins and inhibit the activation of alternative pathway.

OA mouse models have a prominent role in the pathogenesis and therapy aspects. The most used models include surgical models, mechanical loading models, spontaneous and genetic models, chemically-induced models and high-fat dietary and/or obesity models [25]. The chemically-induced models could be obtained rapidly, and the cost is low. However, they were less clinically-relevant compared with other models. High-fat dietary and/or obesity models could mimic the OA caused by specific diets and obesity. However, the period is long, and the cost is high. Spontaneous models could mimic the human OA well, even different stages of OA. Additionally, genetic models were suitable for pathophysiology studies. The disadvantages for spontaneous and genetic models are the long period and high cost. The variability during OA progression was high. Mechanical loading models are used to investigate blunt injury in human knee, while it is not commercially available for now. Surgical models could mimic post-traumatic OA and different stages of OA. The DMM model could mimic the slowly progressing OA, while the anterior cruciate ligament transection (ACLT) model lead to more severe and rapid damage than the DMM model. The reproducibility is high. However, surgical models need strong surgical skills. In this study, the DMM model was used to investigate mild to moderate OA. The mouse strains could affect the DMM model. It is reported that the DMM model with the highest severity is 129/SvEv mouse. The second one is C57BL/6 mouse [26]. In this study C57BL/6 mouse was used to investigate the bioactivities of LMWCS-S-O in vivo.

There are two methods to evaluate the DMM model, including physical characteristics and Safranin-O. The main methods for the physical characteristics are the Von Frey method, the thermal wind-up method and the functional wind-up method [27]. The functional wind-up method was selected to evaluate the pain caused by OA. The Safranin-O method is the most used method to evaluate the severity [28]. Additionally, the semi-quantitative OA grade analysis method was used in this study according to [28,29]. The results showed that the DMM model was successfully established in C57BL/6 mice by the surgical method. In the current study, the DMM model results documented a protective effect of LMWCS-S-O in OA development.

No drug is available to treat OA now, and the drug on the market is used to relieve pain, such as non-steroidal anti-inflammatory drug (NSAID) [25]. The alternative pathway in the complement system plays key roles in OA. The results indicated that LMWCS-S-O could inhibit the formation of C5b-9 in the alternative pathway. Although CS-P could inhibit the alternative pathway, the anti-complement capacity was not as good as LMWCS-S-O. Whether orally-administered LMWCS is delivered to the affected area should be investigated further. However, Ronca et al. [30] and Souich et al. [31] demonstrated that CS administrated orally could reach the joint and distribute into the cartilage and subchondral layers. Additionally, Martel-Pelletier et al. [32] indicated that LMWCS might be more readily absorbed and, hence, more effective. Therefore, LMWCS-S-O was proven to have potential in attenuating OA through regulating the alternative pathway of the complement system.

## 4. Experimental Section

### 4.1. Materials

Bovine, porcine and shark cartilage CSs were purchased from Shandong Kangping Bio Technology Co., Ltd. (Linyi, China), Huamao Shuanghui Co., Ltd. (Luohe, China) and Yantai Dongcheng Co., Ltd. (Yantai, China), separately. CS disaccharides (Δdi-0S, Δdi-4S, Δdi-6S, Δdi-2,6diS and Δdi-4,6diS) and chondroitinase ABC from *Proteus vulgaris* were obtained from Sigma (St. Louis, MO, USA). Mg^2+^-EGTA buffer (2.5 mM Veronal buffer, containing 70 mM NaCl, 140 mM glucose, 0.1% gelatin, 7 mM MgCl_2_, 10 mM EGTA, pH 7.4) was self-prepared. Normal human serum (NHS) was donated by volunteers in our lab. The rabbit blood collected from normal adult rabbit heart was put into Alsever’s solution. The erythrocytes were washed three times with Mg^2+^-EGTA buffer and resuspended in Mg^2+^-EGTA buffer at 4 °C up to 3 days. Mouse terminal complement complex C5b-9 ELISA kit (Cusabio Biotech Co., Ltd., Wuhan, China) was used for the C5b-9 determination. Mouse serum samples were from C57BL/6J mice. HRP-conjugated rabbit anti-mouse C3 antibody was from CUSABIO in China. All other chemical reagents were of analytical grade.

### 4.2. Animals

Male C57BL/6J mice (aged 8–9 weeks), purchased from Vital River Laboratories (Beijing, China, Document No. SCXK 2012-0001), were adapted to standard laboratory conditions (25 ± 3 °C, 55% ± 5% humidity and 12 h light/dark cycle) for 2 weeks. All care and handling of animals were performed with the approval of the Institutional Animal Care and Use Committee of Shandong University (LL201402083, 12 May 2014). The Guidelines of Institutional Animal Ethics Committee were followed for in vivo experiments.

### 4.3. Sample Preparation

The HCl degradation method was introduced to get LMWCS. Three grams of shark CS (CS-S) were dissolved in 150 mL of deionized water. Two-point-five-eight milliliters of HCl (37%, *v*/*v*) were added into the solution and incubated for 10 h at 60 °C with vigorous stirring. The solution was adjusted to pH 7 with NaOH (1 M) and ultrafiltrated with a molecular weight cut-off (MWCO) of a 5000-Da membrane to get the fraction with a relative molecular mass (*M*_r_) lower than 5000 Da. Finally, the fraction was desalted using dialysis tubing (MWCO 500 Da) against deionized water for 24 h, followed by freezing-drying to get the LMWCS from CS-S with the acid degradation method named as LMWCS-S-A. The same methods were performed on bovine CS (CS-B) and porcine CS (CS-P) to get LMWCS-B-A and LMWCS-P-A, respectively.

The enzymatic method was employed to prepare LMWCS. One gram of CS-S in 150 mL of deionized water was incubated with 10 U of chondroitinase for 4 h at 37 °C. After the enzyme was deactivated and removed, the solution was treated using the same steps as above to obtain the LMWCS from CS-S with the enzymatic degradation method, named as LMWCS-S-E. The same methods were performed on CS-B and CS-P to get LMWCS-B-E and LMWCS-P-E, respectively.

The microwave-assisted alkali degradation method was used to prepare LMWCS. Point-four grams of CS-S were dissolved in 20 mL of 0.1 M NaOH solution in a 25-mL flask and then treated with a microwave oven at 60 °C for 10 min. The solution was adjusted to pH 7 and treated using the same steps as mentioned in the LMWCS-S-A preparation to obtain the LMWCS from CS-S with the microwave-assisted alkali degradation method, named as LMWCS-S-M. The same methods were performed on CS-B and CS-P to get LMWCS-B-M and LMWCS-P-M, respectively.

The oxidative degradation method was used to get LMWCS. Three grams of CS-S were dissolved in 100 mL of deionized water, and 20 mL of 30% hydrogen peroxide were added into the solution. The solution was adjusted to pH 5 and incubated at 65 °C for 10 h. The solution was adjusted to pH 7 and treated using the same steps as mentioned in the LMWCS-S-A preparation to obtain the LMWCS from CS-S with the oxidative degradation method, named as LMWCS-S-O. The same methods were performed on CS-B and CS-P to get LMWCS-B-O and LMWCS-P-O, respectively.

### 4.4. Characterization of LMWCS

#### 4.4.1. Disaccharide Composition

One hundred microliters of a 10-mg/mL CS sample in deionized water and 100 μL of chondroitinase ABC (1 U/mL) were added into 800 μL of Tris-HCl buffer (pH 8.0) and incubated at 37 °C for 1 h. The reaction was stopped by boiling the solutions for 5 min, followed by centrifugation at 12,000 rpm for 20 min. The supernatant was filtered by a 0.22-μm filter. The composition of disaccharides was analyzed by strong anion-exchange chromatography on a Waters 2695 HPLC system equipped with a Spherisorb SAX column (250 mm × 4.6 mm, 5 μm; Waters, Milford, MA, USA). The injection volume was 20 μL, and the UV detector was set at 232 nm. The mobile phase consisted of pH 3.5 hydrochloric acid solution (Mobile Phase A) and 2 M sodium chloride solution (pH = 3.5, Mobile Phase B). The elution was isocratic for the first 4 min at 100% of Phase A, changed to 50/50 (*v*/*v*) A/B following 41 min at a flow rate of 1 mL/min. Δdi-0S, Δdi-4S, Δdi-6S, Δdi-2,6diS and Δdi-4,6diS were used as standards to identify the peaks in HPLC chromatography.

#### 4.4.2. Ultraviolet–Visible (UV) Absorption

UV spectra were collected by the Cary 60 spectrometer (Agilent Technologies, Santa Clara, CA, USA), and the range was from 190 nm to 800 nm.

#### 4.4.3. Nuclear Magnetic Resonance (NMR)

NMR analyses were performed with a Bruker Avance 500 spectrometer of 500 MHz in the Fourier transform (FT) mode. All samples were lyophilized and then dissolved in deuterium oxide (D_2_O, 99.9% D) at about 10 mg/mL. NMR spectra were calibrated using the solvent signal (δ 4.79 for D_2_O).

#### 4.4.4. Molecular Weight Determination

The weight-average molecular weight (${\bar{M}}_{W}$) was determined by SEC-MALLS using a Waters 515 HPLC Pump (Waters, Milford, MA, USA) combined with a DAWN EOS 18-angle laser light scattering detector and an OPTILAB refractive index detector (Wyatt Technologies Corp., Goleta, CA, USA). The mobile phase was 0.2 M NaNO_3_. The flow rate was 1 mL/min. The injection volume was 100 μL. The refractive index increment (dn/dc) used for CS was 0.1427. Wyatt ASTRA software Version 5.3 was used to calculate the ${\bar{M}}_{W}$.

### 4.5. In Vitro Anti-Complement Activity Analysis

#### 4.5.1. Hemolytic Analysis

The anti-complement activity for the alternative pathway of LMWCS was determined by hemolytic analysis reported by Servais [33] with a slight modification. Briefly, 10 μL of erythrocytes and 90 μL of deionized water were added into 96-well microplates. Hemolysis of the supernatant was measured at 405 nm after centrifugation for 5 min at 2000 rpm to adjust the absorbance to 1. Ten microliters of NHS and 10 μL of LMWCS solution at different concentrations were mixed with 70 μL of Mg^2+^-EGTA buffer for 15 min on ice. Then, 10 μL of rabbit erythrocytes were added into the solution and incubated for 30 min at 37 °C. After centrifugation for 5 min at 2000 rpm, the absorbance of the supernatant at 405 nm was measured. The IC_50_ was calculated to compare their anti-complement activities.

#### 4.5.2. Anti-C3 Deposition Capacity Determination

The anti-C3 deposition capacity was determined by the ELISA method [34]. One hundred microliters of zymosan (2 × 10^8^ particles/mL) were coated in 96 wells with coating buffer (0.1 M carbonate/bicarbonate buffer, pH 9.5) and incubated overnight at 4 °C. Wells coated with 1% BSA were used as negative controls. All wells were washed for four times with wash buffer (10 mM PBS, pH 7.4, 0.05% Tween 20). Then, 300 μL of blocking buffer (0.1% BSA in 100 mM Tris, pH 7.4, 140 mM NaCl, 10 mM CaCl_2_) were added for incubation at room temperature for 2 h. Different concentrations of LMWCS-S-O and CS-P were pre-incubated with mice serum on ice for 20 min. All samples were transferred into the wells and incubated at 37 °C for 1 h. The wells were rinsed with wash buffer four times, and HRP-conjugated rabbit anti-mouse C3 antibody was added and incubated at room temperature for 1 h. After that, the wells were rinsed; 100 μL of TMB solution were added into each well and incubated for 15–25 min. Fifty microliters of 1 M H_2_SO_4_ were added into each well, and absorbance at 450 nm was recorded.

#### 4.5.3. Cell Viability Analysis

Primary murine chondrocytes were isolated from C57BL/6J mice knee joints and cultured according to the established method [35]. Immature murine articular chondrocytes were identified by the trypan blue and alcian blue methods.

The MTT assay was carried out to measure the cytotoxic effects of LMWCS samples on chondrocyte proliferation. The cells were cultured in 96-well plates at a density of 3 × 10^3^ cells/well in 100 μL of Dulbecco’s Modified Eagle Medium (DMEM) low glucose medium with different concentrations of LMWCS samples for 24 h at 37 °C and 5% CO_2_. Then, the medium was discarded, and 100 μL of MTT solution were added to each well and incubated for 4 h. Finally, the supernatant was discarded, and 150 μL of DMSO were added to each well to dissolve the formazan. The absorbance at 490 nm was determined by the microplate reader after shaking the plate.

Chondrocytes were seeded in a 96-well plate at a density of 2 × 10^4^ cells/well. The cells were cultured with DMEM containing 10% FBS overnight at 37 °C and 5% CO_2_. Culture medium was removed on the second day, and 100 μL of LMWCS-S-O and CS-P in 20% NHS at different concentrations were added and incubated for 12 and 24 h at 37 °C and 5% CO_2_. The cells in the control and model groups were cultured with DMEM containing 10% FBS or 20% human serum. Cell Counting Kit-8 (Yiyuan biotech, Guangzhou, China) was used to determine the viability of the cells.

### 4.6. In Vivo Experiments

#### 4.6.1. Animal Model and Treatment

The OA model was generated by DMM surgery, which was regarded to be similar to human OA [28,29]. Mice were randomly divided into six groups with eight mice per group. Three groups of mice were administered orally by LMWCS-S-O at the doses of 50, 150 and 450 mg/kg, respectively. One group was administered with CS-P at the dose of 150 mg/kg. The OA model group and sham group were administered with an equal volume of saline once a day. The bodyweights were weighed at the first day and 12 weeks.

#### 4.6.2. Functional Wind-up Determination

A modified FWU method [27] was used to measure the supporting force difference of two hind legs after 12 weeks of surgery. A climbing task with a 60 degree angle was performed. When the mouse stood up and started to climb the slope, the supporting force difference between the left and right hind legs was recorded in order to evaluate the pain caused by OA.

#### 4.6.3. C5b-9 Level Determinations and Histological Analysis

At 12 weeks, blood was taken from the mice’s orbital sinus according to previous method [36]. The blood samples were placed at 4 °C for 2 h and then centrifuged at 10,000 rpm for 10 min to get the serum. The ELISA kit was used to detect the levels of C5b-9 in the serum according to the ELISA protocol.

The mice were euthanized and fixed on a plate by fixing the anterior legs with needles. Skin and soft tissues were removed from the right-hind legs using scissors and pincer. The knee joints were taken out by scissors, fixed in 4% paraformaldehyde for 2 days and then decalcified in EDTA for at least 14 days on a shaker. The joints were embedded in paraffin, and 6-μm frontal sections were taken from the entire joint at 80-μm intervals. Finally, the joints were stained with Safranin-O, and 6-point scaling was used to evaluate the OA according to references [25,26].

### 4.7. Statistical Analysis

All data were analyzed using the SPSS18.0 statistical software package (IBM, Armonk, NY, USA). Statistical significance between groups was determined by one-way analysis of variance (ANOVA). *p* < 0.05 was considered to be significant.

## 5. Conclusions

CS and LMWCS from different sources and depolymerization methods have different compositions. This makes CS and LMWCS have different activities. LMWCS-S-O had a higher anti-complement capacity among all samples. The in vivo results demonstrated that oral administration of LMWCS-S-O could attenuate OA. However, the detailed mechanism of CS and LMWCS on attenuating OA needs further investigation, including whether orally-administrated LMWCS is delivered to the affected area, its effect on different complements and pathways, as well as other aspects other than complement pathways.

## Figures and Tables

**Table 1:** Disaccharide compositions and ${\bar{M}}_{W}$ of all samples.

Sample Name	ΔDi-0S (%)	ΔDi-6S (%)	ΔDi-4S (%)	ΔDi-2,6diS (%)	ΔDi-4,6diS (%)	${\bar{M}}_{W}$ (Da)
LMWCS-B-A	25.16	26.16	48.07	–	–	1826
LMWCS-P-A	30.89	27.24	41.25	–	–	2690
LMWCS-S-A	23.34	37.25	28.19	9.30	1.92	1217
LMWCS-B-E	3.78	16.50	78.94	–	–	2583
LMWCS-P-E	2.48	46.88	49.18	–	–	2920
LMWCS-S-E	0.00	60.87	24.38	9.36	5.39	1994
LMWCS-B-M	35.83	22.42	41.22	–	–	8085
LMWCS-P-M	16.01	29.94	53.37	–	–	3662
LMWCS-S-M	43.97	29.74	18.36	7.52	0.41	4269
LMWCS-B-O	4.86	25.77	68.78	–	–	1561
LMWCS-P-O	4.26	26.87	68.60	–	–	2191
LMWCS-S-O	9.22	50.02	27.50	12.17	1.08	1511
CS-B	7.30	23.54	68.82	–	–	18,150
CS-P	0.88	32.12	66.68	–	–	10,070
CS-S	0.00	54.77	31.31	12.66	1.26	31,300

**Table 2:** Anti-complement capacity of chondroitin sulfates (CSs) from three sources and the depolymerized derivatives.

Sample	Line Equation	IC_50_ (mg)
LMWCS-B-A	y = 0.0967x + 0.7583 *R* = 0.989	5.59
LMWCS-P-A	y = 0.11x + 1.8179 *R* = 0.989	7.32
LMWCS-S-A	y = 0.0703x + 1.2167 *R* = 0.9599	4.76
LMWCS-B-E	y = 0.0703x + 0.8147 *R* = 0.980	4.33
LMWCS-P-E	y = 0.064x + 1.8861 *R* = 0.992	5.09
LMWCS-S-E	y = 0.0589x + 1.2021 *R* = 0.977	4.15
LMWCS-B-M	y = 0.1016x − 1.3729 *R* = 0.984	3.71
LMWCS-P-M	y = 0.0613x + 1.5563 *R* = 0.994	4.62
LMWCS-S-M	y = 0.0815x + 1.1212 *R* = 0.991	5.20
LMWCS-B-O	y = 0.0891x + 0.3776 *R* = 0.994	4.83
LMWCS-P-O	y = 0.0573x + 1.425 *R* = 0.975	4.29
LMWCS-S-O	y = 0.0565x + 0.5873 *R* = 0.988	3.41
CS-B	y = 0.1029x + 1.0596 *R* = 0.993	6.20
CS-P	y = 0.1499x + 1.5709 *R* = 0.988	9.07
CS-S	y = 0.0902x − 0.0251 *R* = 0.987	4.48
